# Brain insulin resistance and microglial immunometabolic dysregulation in neurodegenerative diseases

**DOI:** 10.3389/fnagi.2026.2002283

**Published:** 2026-10-26

**Authors:** Xinyu Liu, Xiaorong Zhang, Yufang Su, Gaopeng Guan, Xianjun Luo, Puhua Zhang

**Affiliations:** 1 Department of Internal Medicine, Clinical Medical School of Jiujiang University, Jiujiang, China; 2 Jiujiang Clinical Precision Medicine Research Center, Affiliated Hospital of Jiujiang University, Jiujiang, China; 3 Department of Oncology, Jiangxi Provincial Women and Children’s Medical Center, Nanchang, China; 4 Department of Endocrinology, Central South University Third Xiangya Hospital, Changsha, China

**Keywords:** Alzheimer disease, brain insulin resistance, microglia, neurodegenerative diseases, NLRP3 inflammasome, Parkinson disease, persistent inflammation

## Abstract

Brain insulin resistance (BIR) is increasingly recognized as a contributor to the development and progression of neurodegenerative diseases rather than simply a metabolic abnormality. In the central nervous system, insulin signaling is involved in neuronal survival, synaptic plasticity, energy metabolism, and protein homeostasis. Increasing evidence suggests that impaired brain insulin signaling is associated with Alzheimer disease (AD), Parkinson disease (PD), and other neurodegenerative conditions, although the mechanisms linking BIR to persistent inflammation and neuronal injury remain incompletely understood. Microglia, the principal innate immune cells of the central nervous system, integrate metabolic and immune signals and may therefore provide an important link between impaired insulin signaling and chronic inflammation. BIR may disrupt insulin receptor substrate (IRS), phosphoinositide 3-kinase (PI3K), and protein kinase B (Akt) signaling and promote nuclear factor-kappa B (NF-κB) activation, mitochondrial dysfunction, oxidative stress, impaired mitophagy, and lysosomal and lipid metabolic disturbances. These changes may promote activation of the NLR family pyrin domain-containing 3 (NLRP3) inflammasome, inflammatory mediator release, excessive phagocytosis, and synaptic dysfunction. In turn, persistent microglial inflammation may further impair insulin signaling, forming a reciprocal feedback loop that sustains pathological changes. This review summarizes the mechanisms linking BIR to microglial immunometabolic dysfunction and discusses potential therapeutic strategies targeting metabolic and inflammatory pathways in neurodegenerative diseases.

## 1 Introduction

Neurodegenerative diseases are a group of chronic disorders characterized by progressive damage, functional decline, and irreversible loss of specific neuronal populations in the central nervous system (Liu et al., 2019). Among them, Alzheimer disease (AD) and Parkinson disease (PD) are the most representative. AD is characterized by progressive cognitive decline accompanied by pathological features such as β-amyloid deposition and tau abnormalities (Jia et al., 2025). In contrast, PD is primarily characterized by the loss of dopaminergic neurons in the substantia nigra and α-synuclein related pathology (Ye et al., 2023). With accelerated population aging, the prevalence of neurodegenerative diseases continues to rise, making them a major public health challenge that severely affects quality of life and imposes a substantial socioeconomic burden.

In recent years, increasing attention has been directed toward the role of brain insulin resistance in the onset and progression of neurodegenerative diseases. In the central nervous system, insulin signaling is involved not only in energy metabolism but also in neuronal survival, synaptic plasticity, and cognitive maintenance. Current evidence suggests that brain insulin resistance (BIR) is associated with the pathological progression of neurodegenerative diseases such as AD and PD and may contribute to abnormal protein metabolism, synaptic dysfunction, and inflammatory responses (Chen et al., 2022; Biessels et al., 2020; Wakabayashi et al., 2019).

Microglia are the principal innate immune cells of the central nervous system. In addition to immune surveillance and responses to tissue injury, they participate in synaptic homeostasis, clearance of abnormal proteins, and regulation of local metabolism. Their functional state is highly sensitive to changes in the brain microenvironment (Asimakidou et al., 2025; Sun and Mi, 2025). Insulin signaling abnormalities associated with BIR may affect microglial energy metabolism, mitochondrial function, lipid handling, and phagocytic degradative capacity, while also altering their responsiveness to injury-related signals. As a result, microglia may shift from a homeostatic state toward persistent immune activation and contribute to neuronal and synaptic injury through the release of inflammatory mediators, oxidative stress, and impaired clearance of abnormal proteins.

Several previous reviews have examined brain insulin resistance across neuronal and glial cell types, whereas others have focused primarily on microglial activation, immunometabolism, or neuroinflammation in neurodegenerative disease. More recent work has begun to establish a direct role for microglial insulin signaling in experimental AD models. However, the evidence connecting BIR to microglial immunometabolic dysfunction remains heterogeneous across cell types, diseases, and experimental systems. The present review therefore focuses specifically on the proposed BIR–microglia–inflammation axis, while distinguishing direct cell-specific evidence from mechanistic inference, comparing shared and disease-specific mechanisms across neurodegenerative disorders, and evaluating the relative strength and translational stage of therapeutic evidence (Sun and Mi, 2025; Chen et al., 2025; Xiang et al., 2026).

In this review, we propose that BIR should be viewed not only as a neuronal metabolic defect but also as a driver of microglial immunometabolic reprogramming that sustains chronic neuroinflammation. We first summarize the physiological roles of insulin signaling in the central nervous system and the mechanisms leading to BIR. We then discuss microglial homeostatic functions, activation states, and disease-associated phenotypes, with particular emphasis on their capacity to integrate metabolic and immune signals. The central part of this review focuses on the molecular mechanisms by which impaired insulin signaling promotes persistent microglial inflammation, including insulin receptor substrate (IRS), phosphoinositide 3-kinase (PI3K), and protein kinase B (Akt) signaling dysfunction, nuclear factor-kappa B (NF-κB) activation, mitochondrial damage, oxidative stress, impaired mitophagy, lysosomal and lipid metabolic stress, NLR family pyrin domain-containing 3 (NLRP3) inflammasome activation, complement-mediated synaptic injury, and feed-forward inflammatory loops. Finally, we compare the shared and disease-specific mechanisms linking BIR and microglial dysfunction across neurodegenerative disorders, evaluate the relative strength and translational stage of the available evidence, and discuss therapeutic strategies and emerging hypotheses for interrupting the BIR–microglia–inflammation axis.

### 1.1 Literature identification and evidence appraisal

Relevant peer-reviewed literature was identified using combinations of keywords related to brain insulin resistance, insulin signaling, microglia, immunometabolism, neuroinflammation, synaptic dysfunction, and neurodegenerative diseases, followed by screening of relevant reference lists. Recent mechanistic, experimental, and clinical studies were prioritized, while seminal studies were retained when necessary to provide the conceptual or mechanistic basis for established pathways. During synthesis, evidence was interpreted according to study type, cell-type specificity, disease context, and translational stage. Particular attention was given to distinguishing direct evidence for insulin signaling dysfunction in microglia from findings inferred from neurons, astrocytes, peripheral metabolic tissues, or non-cell-specific models.

## 2 Brain insulin signaling and the development of brain insulin resistance

### 2.1 Physiological functions of insulin signaling in the central nervous system

Insulin has long been regarded primarily as a peripheral metabolic hormone that regulates blood glucose homeostasis, lipid metabolism, and protein synthesis. However, accumulating evidence indicates that the brain is also an important target organ of insulin (Heni, 2024). Brain insulin is derived mainly from the peripheral circulation and enters the central nervous system through selective and saturable transport across endothelial cells of the blood–brain barrier (Zhu et al., 2025). Although it remains controversial whether insulin is synthesized locally in the brain (Arnold et al., 2018), insulin signaling plays important physiological roles in regions such as the hippocampus, hypothalamus, cortex, olfactory bulb, and striatum, regardless of its source.

In the brain, insulin acts primarily through the insulin receptor and insulin-like growth factor receptors. Binding of insulin to the insulin receptor induces autophosphorylation of the receptor β subunit and recruitment of insulin receptor substrates 1 and 2, thereby activating the PI3K and Akt signaling pathway (Martínez Báez et al., 2024). The PI3K and Akt pathway is one of the major signaling cascades in brain insulin signaling and participates in the regulation of glucose uptake, cell survival, synaptic plasticity, protein synthesis, and anti-inflammatory responses (Huang et al., 2018). Downstream effectors of Akt, including GSK3β, mTOR, FOXO, and Nrf2, are closely linked to neurodegenerative diseases (Kumari et al., 2023). Under physiological conditions, Akt inhibits GSK3β through phosphorylation, thereby helping maintain stable tau phosphorylation and normal synaptic function (Chu et al., 2017). mTOR regulates protein synthesis, autophagy, and synaptic plasticity, and dysregulation of its activity may disrupt protein homeostasis and cellular metabolic balance (Rejili et al., 2026). Taken together, the PI3K and Akt pathway and its downstream effectors form the core signaling network through which insulin exerts metabolic support and neuroprotective effects in the brain.

In addition to PI3K and Akt, insulin signaling also regulates cell proliferation, differentiation, neural plasticity, and immune responses through the MAPK and ERK pathway. These two canonical pathways act together to maintain normal central nervous system function (He et al., 2025). Importantly, the actions of insulin in the brain extend beyond neurons. Recent studies indicate that insulin receptors are expressed in several non-neuronal cell types, including astrocytes, microglia, and cerebrovascular endothelial cells, although receptor expression and functional responsiveness may vary across cell types and experimental contexts (Porniece Kumar et al., 2021; Chen et al., 2023). In particular, insulin signaling in microglia plays an important role in the regulation of cellular energy metabolism, phagocytic function, and inflammatory phenotype (Nicholson et al., 2026). Thus, normal brain insulin signaling is essential not only for neuronal metabolism and function but also for neurovascular homeostasis and neuroimmune regulation. This concept provides an important basis for understanding how metabolic disorders perturb the central nervous system microenvironment.

### 2.2 Molecular mechanisms leading to brain insulin resistance

Because insulin signaling contributes to the maintenance of the central metabolic and immune microenvironment, prolonged metabolic stress can progressively impair this system and lead to BIR. BIR is defined as a reduced responsiveness of the central nervous system to insulin stimulation and is primarily manifested as attenuated insulin receptor signaling and suppression of the downstream PI3K and Akt pathway (Arvanitakis et al., 2021). The development of this pathological state reflects the interaction of multiple factors. On the one hand, hyperglycemia, high fat exposure, and systemic inflammation associated with obesity and type 2 diabetes can affect the brain and trigger central metabolic stress. On the other hand, aging, local oxidative stress, and neurodegenerative pathological factors such as β-amyloid, abnormal tau, and α-synuclein can further disrupt insulin signaling within the brain (Bentivegna et al., 2025; de la Monte, 2017).

At the molecular level, multiple metabolic and stress-related factors can converge on and disrupt the insulin receptor, IRS, PI3K, and Akt signaling axis. High glucose, excess lipids, inflammatory mediators, reactive oxygen species, and abnormal protein aggregates may all impair IRS function, whereas abnormalities in negative regulators such as SOCS and PTP1B may further weaken insulin signaling (Lebrun and Van Obberghen, 2008). In addition, peripheral hyperinsulinemia may downregulate insulin transport across the blood–brain barrier, further reducing central insulin sensitivity (Asimakidou et al., 2025; Heni et al., 2015). Thus, BIR does not arise from a single molecular defect but from the combined effects of metabolic, inflammatory, and neurodegenerative factors on the central insulin signaling network.

These consequences provide an important basis for considering microglia as a cellular link between impaired insulin signaling and central immune responses. The following section therefore focuses on the physiological and disease-associated states of microglia that provide the cellular context for BIR-related neuroinflammation.

## 3 Microglia as immunometabolic sensors in the central nervous system

### 3.1 Homeostatic functions of microglia

Microglia originate from embryonic yolk sac myeloid progenitors and are the resident macrophage-like innate immune cells of the central nervous system (Shimizu and Prinz, 2025; Prinz et al., 2019; Ginhoux et al., 2010). Unlike peripheral monocyte derived macrophages, microglia undergo long-term self-renewal within the brain and are continuously shaped by signals from the local microenvironment (Kong et al., 2026; Askew et al., 2017; Escoubas and Molofsky, 2024). Under physiological conditions, microglia possess highly dynamic processes that enable them to continuously survey the surrounding environment and adapt their functional state in response to local cues.

Microglia express a diverse array of pattern recognition receptors, chemokine receptors, complement receptors, and purinergic receptors, allowing them to detect pathogens, tissue damage, and local metabolic changes (Portugal et al., 2022; Paulson et al., 2026; Kettenmann et al., 2011). Under homeostatic conditions, they participate in the clearance of apoptotic cells and abnormal proteins and contribute to synaptic pruning through complement related mechanisms. They also release neurotrophic factors such as BDNF and IGF 1, thereby supporting neuronal survival and synaptic plasticity (Cornell et al., 2022). Therefore, microglia are not merely defensive immune cells. They are also central regulators of immune surveillance, molecular clearance, synaptic homeostasis, and neuronal support in the brain. Their high functional plasticity enables rapid adaptation to metabolic and pathological stimuli, but also makes them important pathological effector cells in neurodegenerative diseases.

### 3.2 Microglial heterogeneity and disease-associated phenotypes

Traditional studies often classified microglial activation into two categories, namely M1 and M2. The M1 phenotype is generally associated with the release of pro-inflammatory mediators such as TNF-α, IL-1β, IL-6, nitric oxide, and reactive oxygen species, whereas the M2 phenotype is more closely associated with IL-10, TGF-β, and tissue repair (Tang and Le, 2016). However, with the development of single cell RNA sequencing, spatial transcriptomics, and related technologies, increasing evidence indicates that microglial functional states are highly continuous and heterogeneous and cannot be fully captured by the simple M1 and M2 framework (Cheng and Ho, 2025; Chen and Colonna, 2021).

During aging and in neurodegenerative diseases, microglia can adopt diverse disease-associated states. Among these, disease-associated microglia have been identified in models of AD, amyotrophic lateral sclerosis, and aging (Chen et al., 2024; Keren-Shaul et al., 2017). Disease-associated microglia are typically characterized by reduced expression of homeostatic genes such as P2ry12, Tmem119, and Cx3cr1, together with increased expression of disease-associated genes including Apolipoprotein E (APOE), Triggering receptor expressed on myeloid cells 2(TREM2), CST7, LPL, and TYROBP, accompanied by marked remodeling of lipid metabolism and phagocytic function (Chen and Colonna, 2021). The functional role of disease-associated microglia is strongly stage dependent. In early disease stages, enhanced phagocytic and lipid processing capacities may facilitate the clearance of abnormal proteins and cellular debris (Keren-Shaul et al., 2017; Sha et al., 2025). However, under persistent exposure to pathological stimuli, including β-amyloid, α-synuclein aggregates, extracellular ATP and HMGB1, oxidative and lipid stress, and sustained pro-inflammatory signals such as TNF-α and IL-1β, certain disease-associated states may exhibit reduced phagocytic capacity and increased release of inflammatory mediators, thereby promoting neuronal injury (Chen et al., 2026; Marschallinger et al., 2020). In addition, the microglial neurodegenerative phenotype further suggests that the disease environment can profoundly reprogram homeostatic transcriptional programs and the metabolic state of microglia (Krasemann et al., 2017), a process closely linked to signaling axes such as TREM2 and APOE.

Importantly, disease-associated microglia and the microglial neurodegenerative phenotype reflect more than changes in immune phenotype alone. They represent integrated adaptations of microglia to sustained pathological stress, including remodeling of lipid metabolism, energy utilization, phagocytosis, and organelle homeostasis. Thus, the immune and metabolic states of microglia are tightly coupled rather than independent. These features provide a cellular context for examining how impaired insulin signaling may interact with microglial immunometabolic states in neurodegenerative disease.

## 4 Brain insulin resistance-driven microglial neuroinflammation

BIR affects not only neuronal energy metabolism but also neuronal and synaptic remodeling. Impaired IRS–PI3K–Akt signaling may alter downstream effectors such as GSK3β, mTOR, and FOXO, thereby disrupting synaptic plasticity, receptor trafficking, protein homeostasis, and autophagic regulation. Experimental studies have shown that brain insulin resistance can impair hippocampal long-term potentiation and memory through altered FoxO3a-dependent regulation of GluA1 palmitoylation, illustrating how impaired insulin signaling can be translated into functional synaptic remodeling (Spinelli et al., 2017). In parallel, altered insulin signaling may increase neuronal vulnerability to oxidative and metabolic stress and compromise the maintenance of synaptic and proteostatic homeostasis. These neuronal changes occur alongside, rather than independently of, alterations in microglial immunometabolism. When insulin signaling is impaired, microglia may become more sensitive to metabolic stress, abnormal proteins, and injury-related cues, while their energy metabolism, organelle quality control, and inflammatory regulation may be compromised (Sun and Mi, 2025; Yang et al., 2022).

### 4.1 Impaired insulin signaling promotes microglial pro-inflammatory susceptibility

Microglia are not only effector cells of neuroinflammation but also important targets of insulin signaling (Chen et al., 2025). Previous studies have shown that microglia express insulin receptors and downstream signaling molecules, including IRS-1, IRS-2, PI3K, Akt, GSK3β, and mTOR, suggesting that insulin may directly regulate microglial metabolism, inflammatory responsiveness, and phagocytic function (Chen et al., 2025; Barone and Butterfield, 2025). Therefore, under conditions of brain insulin resistance, microglial dysfunction may involve not only altered production of inflammatory mediators but also broader disturbances in energy metabolism, organelle homeostasis, and inflammatory threshold regulation.

Evidence for impaired insulin signal transduction in microglia is emerging, although the molecular mechanisms remain incompletely defined. Recent microglia-specific experimental evidence indicates that loss of insulin receptor signaling can induce metabolic reprogramming, alter innate immune pathways, impair Aβ uptake, and exacerbate neuroinflammation in an AD model (Chen et al., 2025). At the molecular level, impaired insulin signaling may involve inhibitory serine phosphorylation of IRS proteins, which can weaken IRS-dependent coupling to PI3K and thereby attenuate downstream Akt signaling (Copps and White, 2012; Bassil et al., 2017). Stress-responsive kinases, including JNK, IKKβ, PKC, and mTOR/S6K, have been implicated in the regulation of IRS serine phosphorylation and insulin resistance in other metabolic or cellular contexts (Tanti and Jager, 2009; Yuan et al., 2016; Woo et al., 2024). However, direct evidence establishing these kinase-mediated mechanisms specifically in microglia remains limited, and their precise contribution to microglial insulin resistance requires further validation.

Importantly, serine phosphorylation of IRS proteins is not uniformly pathological. Some phosphorylation sites are involved in physiological negative feedback regulation, and their functional consequences depend on the specific phosphorylation site, upstream kinase, and cell type (Zeke et al., 2022). At present, direct evidence for IRS serine phosphorylation in microglia remains limited, and much of the current understanding is extrapolated from peripheral metabolic tissues. Therefore, the key IRS phosphorylation sites and upstream regulatory mechanisms in central innate immune cells require further validation.

Disruption of the IRS/PI3K/Akt pathway may therefore increase the inflammatory susceptibility of microglia (Sun and Mi, 2025). Akt is a central node linking insulin signaling to metabolic and inflammatory regulation. Reduced Akt activity weakens inhibitory phosphorylation of GSK3β, thereby enhancing GSK3β activity and facilitating the transcriptional activation of pro-inflammatory genes, including TNF-α, IL-1β, and IL-6 (Wang et al., 2022). Meanwhile, attenuated Akt signaling may affect downstream regulators such as FOXO, mTOR, and Nrf2, leading to impaired antioxidant defense, altered autophagic regulation, and reduced stress adaptation (Cuadrado et al., 2019). Together, these changes extend beyond inflammatory signaling itself and may compromise the metabolic and stress-adaptive functions of microglia. These changes increase the metabolic burden on mitochondrial, lysosomal, and lipid-processing systems and render microglia more vulnerable to inflammatory stimuli.

Thus, impaired insulin signaling in microglia should be understood as a broader decline in immunometabolic regulatory capacity rather than as a defect in a single inflammatory pathway. By weakening Akt-dependent metabolic, antioxidant and anti-inflammatory control, brain insulin resistance may lower the threshold for microglial activation and increase their susceptibility to subsequent inflammatory stimuli. NF-κB represents one major pathway through which this heightened inflammatory susceptibility can be translated into sustained pro-inflammatory activation.

### 4.2 NF-κB activation as an inflammatory priming mechanism in insulin-resistant microglia

The NF-κB pathway plays a central role in the pro-inflammatory transition of microglia (Guo et al., 2024). Under conditions of brain insulin resistance, multiple stimuli, including free fatty acids, advanced glycation end products, β-amyloid, α-synuclein, reactive oxygen species, and damage-associated molecular patterns, can converge to activate NF-κB signaling (Dutta et al., 2021; Hughes et al., 2020). These stimuli are sensed by receptors such as TLR2, TLR4, and RAGE, which activate downstream signaling molecules including MyD88, IRAK, TRAF6, and the IKK complex. Subsequent degradation of IκBα allows NF-κB p65/p50 to translocate into the nucleus and initiate the transcription of pro-inflammatory mediators such as TNF-α, IL-1β, IL-6, and enzymes involved in oxidative and nitrosative stress responses (Zhou et al., 2024).

Under physiological conditions, insulin signaling can partially restrain excessive TLR/NF-κB activation and thereby maintain immune homeostasis in microglia (Chen et al., 2025; Barone and Butterfield, 2025). However, when insulin signaling is impaired, this regulatory effect is weakened, making microglia more prone to inflammatory priming and persistent activation. In this context, NF-κB activation serves as a molecular bridge between impaired insulin signaling and downstream inflammatory effector pathways. It not only promotes the expression of cytokines and chemokines but also increases the expression of inflammasome-related molecules, thereby creating a permissive state for subsequent inflammatory amplification.

It should be noted that the pro-inflammatory transition discussed here is not equivalent to classical M1 polarization. The M1/M2 framework is insufficient to describe the complex and dynamic microglial states observed during aging, metabolic stress, and neurodegenerative disease (Eggen and Kooistra, 2026; Paolicelli et al., 2022). Rather than representing a fixed polarization state, NF-κB-driven activation under brain insulin-resistant conditions should be viewed as a component of persistent immune remodeling within a metabolically disturbed and injury-associated microenvironment.

This sustained NF-κB activation is shaped by both intrinsic and extrinsic factors. Intrinsically, impaired insulin signaling may reduce microglial energy metabolic flexibility, mitochondrial function, lipid processing, and phagocytic-degradative capacity, thereby lowering the tolerance of microglia to pathological stressors (Sun and Mi, 2025; Barone and Butterfield, 2025). Extrinsically, damage-associated molecular patterns such as ATP, HMGB1, mitochondrial DNA, and abnormal protein aggregates released from injured neurons, astrocytes, and cerebrovascular units can continuously stimulate microglia through receptors such as TLRs and RAGE (Picca et al., 2020; Ma et al., 2025). Therefore, disturbances in intrinsic immunometabolic homeostasis and continuous extrinsic danger signals jointly sustain NF-κB activation and maintain microglia in a chronically primed inflammatory state.

### 4.3 Oxidative stress and mitochondrial dysfunction mediate inflammatory amplification

Within the microglial immunometabolic imbalance induced by BIR, mitochondrial dysfunction and oxidative stress are key links between abnormal insulin signaling and persistent inflammation (Barone and Butterfield, 2025; Shah and Doshi, 2026). Under BIR conditions, attenuated IRS, PI3K, and Akt signaling can impair microglial energy metabolism and antioxidant regulatory capacity. Under physiological conditions, pathways such as PI3K and Akt, AMP-activated protein kinase (AMPK), Sirtuin 1 (SIRT1), and Peroxisome proliferator-activated receptor gamma coactivator-1α (PGC-1α) collectively regulate mitochondrial biogenesis, oxidative phosphorylation, and redox homeostasis (Barone and Butterfield, 2025). When these regulatory processes are disrupted, mitochondrial respiratory efficiency declines and electron leakage increases, thereby promoting reactive oxygen species production (Park et al., 2021; Nair et al., 2019). Mitochondrial dysfunction therefore reflects not only impaired energy supply but also a shift in the intracellular redox state of microglia.

Importantly, persistent accumulation of reactive oxygen species may coexist with inadequate clearance of damaged mitochondria. Under normal conditions, PINK1 and Parkin mediated mitophagy recognizes and eliminates damaged mitochondria, thereby limiting sustained release of reactive oxygen species and mitochondria derived danger signals (Pickrell and Youle, 2015). Under insulin resistant conditions, decreased AMPK activity, dysregulated mTOR signaling, and impaired autophagic flux may collectively weaken mitochondrial quality control, leading to progressive accumulation of damaged mitochondria (Herzig and Shaw, 2018; Hsu et al., 2022). Accordingly, persistent oxidative stress in microglia may arise not only from increased reactive oxygen species generation but also from inefficient removal of damaged organelles.

Persistently elevated reactive oxygen species can further impair insulin signaling. Reactive oxygen species activate stress-related kinases such as JNK, IKKβ, and p38 MAPK, which promote abnormal IRS serine phosphorylation and further weaken the functional coupling among IRS, the insulin receptor, and PI3K (Woo et al., 2024; Zeke et al., 2022). This reciprocal relationship between impaired insulin signaling and oxidative stress makes it increasingly difficult for microglia to restore a normal metabolic state, thereby contributing to the persistence of inflammation.

In addition to directly causing oxidative damage, dysfunctional mitochondria also constitute an important source of endogenous immune stimulation. Mitochondria derived molecules such as mitochondrial DNA and cardiolipin can be recognized by the innate immune system after entering the cytoplasm, thereby further enhancing microglial inflammatory responses (Chen et al., 2025; El-Hamri and Abboussi, 2026; Zhu et al., 2025). In this way, mitochondrial damage converts intracellular metabolic abnormalities into immunologically detectable danger signals and provides a crucial basis for subsequent inflammatory amplification.

Mitochondrial dysfunction may also compromise the lipid processing capacity of microglia. During the clearance of myelin debris, β-amyloid, and synaptic structures, microglia must process large amounts of membrane lipids, a task that depends on lipid sensing and lipid handling mechanisms involving molecules such as TREM2 and APOE (Zhu et al., 2025; Takalo et al., 2025; Chausse et al., 2021; Wang et al., 2026). When fatty acid oxidation is reduced and lysosomal degradation is simultaneously impaired, inadequately processed lipids may accumulate as lipid droplets, thereby increasing intracellular lipid burden (Qin et al., 2022). Lipid droplet accumulation may in turn aggravate lysosomal stress, oxidative stress, and phagocytic dysfunction (Qin et al., 2021). Therefore, mitochondrial dysfunction and disruption of lysosomal lipid homeostasis should not be viewed as independent phenomena but rather as interrelated manifestations of declining organelle quality control in microglia.

In the pathological microenvironment of neurodegenerative diseases such as AD and PD, β-amyloid, abnormal tau, and aggregated α-synuclein can further exacerbate these processes. β-amyloid can interfere with the mitochondrial respiratory chain, abnormal tau can disrupt mitochondrial transport, and aggregated α-synuclein may impair mitochondrial dynamics (Sun and Mi, 2025; Akhtar et al., 2024; Peng et al., 2024; Wang et al., 2020). These extracellular pathological stimuli, together with intrinsic metabolic abnormalities in microglia, make organelle damage increasingly difficult to repair.

Thus, BIR-associated mitochondrial, lipid, and lysosomal abnormalities are best understood as interconnected disturbances in organelle homeostasis rather than isolated pathological events. Their combined effect is a sustained increase in reactive oxygen species, mitochondrial DNA, and other intracellular danger signals, which predisposes microglia to persistent inflammatory activation and creates the conditions necessary for subsequent inflammasome related responses.

### 4.4 NLRP3 inflammasome activation as a convergent inflammatory platform

The NLRP3 inflammasome is a key molecular platform in BIR-associated microglial neuroinflammation. Its importance lies in its ability to integrate diverse intracellular and extracellular signals, including oxidative stress, mitochondrial damage, abnormal protein aggregation, and lysosomal injury, and translate them into the release of IL-1β and IL-18 as well as inflammatory cell death (Barone and Butterfield, 2025; Yao et al., 2023; Possemato et al., 2023; Rheinheimer et al., 2017; Xu et al., 2025; Heneka et al., 2018). NLRP3 activation may therefore represent an important convergence point linking metabolic homeostatic imbalance to sustained inflammation.

The NLRP3 inflammasome is composed mainly of NLRP3, ASC, and pro-caspase-1, and its activation requires the coordinated action of inflammatory priming and intracellular activation signals. The priming step is largely associated with NF-κB mediated transcriptional regulation. High fat exposure, β-amyloid, and other pathological stimuli in the BIR microenvironment can promote sustained NF-κB activation, thereby increasing expression of precursor molecules such as NLRP3 and pro-IL-1β and sensitizing microglia to subsequent danger signals (Heneka et al., 2018; Lee et al., 2019; Albornoz et al., 2023; Wei et al., 2025). As described in Section 4.2, this priming process is linked to TLR, RAGE, and NF-κB activation and shifts microglia from a state of inflammatory susceptibility to one permissive for inflammasome assembly.

By contrast, disturbances in mitochondrial and lysosomal homeostasis may provide the intracellular danger signals required for NLRP3 activation. Persistent accumulation of mitochondrial reactive oxygen species and cytosolic mitochondrial DNA, as well as lysosomal damage and cathepsin release associated with lipid overload, may all contribute to NLRP3 inflammasome activation (Lee et al., 2019; Albornoz et al., 2023; Zhang et al., 2024; Muñoz-Planillo et al., 2013). Thus, NLRP3 is not an inflammatory pathway independent of metabolic dysfunction, but rather a key effector platform at which damage signals from multiple organelles converge within microglia.

Once assembled, the inflammasome promotes autocleavage of pro-caspase-1 into enzymatically active caspase-1. Caspase-1 performs two principal cleavage events. It processes pro-IL-1β and pro-IL-18 into their mature pro-inflammatory forms (Rheinheimer et al., 2017; Xu et al., 2025), and it cleaves gasdermin-D, releasing its pore forming N-terminal domain. The N-terminal fragment of gasdermin-D translocates to the plasma membrane, where it oligomerizes and forms pores, thereby potentially inducing pyroptosis (He et al., 2020; Ji et al., 2026; Shi et al., 2015). This process not only results in the release of mature inflammatory cytokines but may also be accompanied by leakage of intracellular damage associated molecular patterns, further stimulating neighboring cells and amplifying local inflammation. Inflammatory mediators such as IL-1β can also disrupt synaptic plasticity, impair astrocyte function, contribute to cognitive dysfunction, and induce astrocytes to adopt neurotoxic phenotypes (Lopez-Rodriguez et al., 2021).

Notably, NLRP3 mediated inflammatory signaling may also feed back onto insulin signaling. IL-1β can promote abnormal IRS serine phosphorylation through stress-related pathways such as JNK and p38, thereby further attenuating PI3K and Akt signaling (He et al., 2020). This mechanism provides a molecular basis for the bidirectional relationship between BIR and chronic inflammation. However, much of the current evidence derives from peripheral metabolic syndrome models, and the extent to which this mechanism operates in the central nervous system, where neurons and glial cells interact in complex networks, remains to be determined.

In AD, microglial phagocytosis of β-amyloid oligomers can impose a persistent lysosomal burden that further promotes NLRP3 activation (Wei et al., 2025; Heneka et al., 2013), potentially establishing a self reinforcing relationship between abnormal protein clearance and inflammasome activation. In PD, aggregated α-synuclein may likewise activate inflammatory signaling in microglia and facilitate the propagation of inflammation to dopaminergic neurons (Lee et al., 2019; Albornoz et al., 2026).

Overall, the role of the NLRP3 inflammasome in BIR-associated neuroinflammation is best viewed as a convergence point for metabolic stress, organelle damage, and inflammatory effector mechanisms. On the one hand, impaired insulin signaling and defective organelle quality control provide the basis for NLRP3 activation. On the other hand, the inflammatory mediators and cell death related signals generated downstream of NLRP3 further exacerbate local tissue injury and sustain the inflammatory response.

Although the NLRP3 inflammasome is widely regarded as a central hub in BIR-associated chronic neuroinflammation, its causal role should be interpreted cautiously. First, much of the available evidence relies on systemic knockout models or broad spectrum inhibitors such as MCC950, and the relative contributions of NLRP3 activation in central microglia and peripheral immune cells remain unclear (Yao et al., 2023; Tejera et al., 2019; Dong et al., 2021). Second, the major activation signals for NLRP3, including potassium efflux, lysosomal rupture, and mitochondrial reactive oxygen species, are highly interconnected at the intracellular level, and no entirely brain-specific direct upstream regulators of NLRP3 have yet been identified (Yao et al., 2023; Shippy et al., 2025). Third, evidence that IL-1β negatively regulates the IRS, PI3K, and Akt pathway still largely derives from peripheral metabolic syndrome models. Whether this negative feedback exerts comparable effects within the complex neuron and glia network of the central nervous system requires direct *in vivo* verification (Rheinheimer et al., 2017; He et al., 2020; Liu et al., 2022; Wu et al., 2021). At present, it is therefore more appropriate to regard NLRP3 as a key convergence point linking metabolic imbalance and chronic inflammation rather than a single disease initiating factor. Together, these mechanisms form a major pathological framework for metabolic stress driven persistent inflammation in microglia, as illustrated in Figure 1. Future therapeutic strategies will likely require not only microglia specific and time dependent modulation of NLRP3 but also simultaneous alleviation of upstream metabolic stress and restoration of organelle homeostasis, thereby interrupting the pathological interplay between BIR and chronic neurodegeneration.

**Figure 1:**
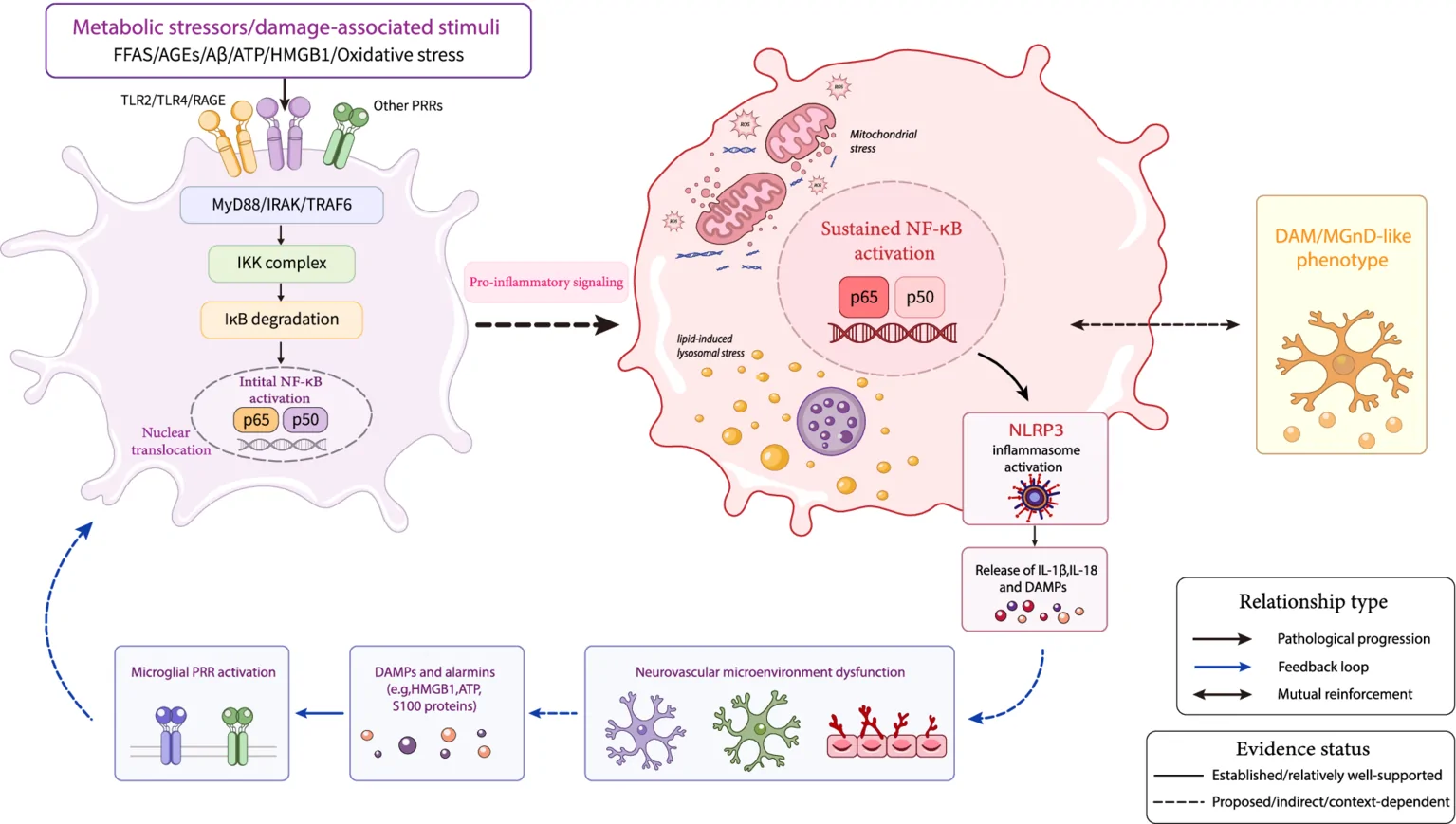
Mechanistic model of sustained microglial inflammatory activation under metabolic stress. Metabolic and damage-associated stimuli, including FFAs, AGEs, Aβ, ATP, HMGB1, and oxidative stress, can engage microglial pattern-recognition receptors and activate the NF-κB signaling cascade through adaptor molecules and the IKK complex, leading to NF-κB activation and inflammatory priming. Sustained inflammatory signaling is accompanied by mitochondrial and lysosomal stress, NLRP3 inflammasome activation, and a DAM/MGnD-like phenotype. The resulting release of IL-1β, IL-18, and DAMPs contributes to neurovascular microenvironment dysfunction and may further stimulate microglial PRRs, forming a feedback loop that sustains inflammatory activation. Bidirectional interactions between sustained NF-κB activation and the DAM/MGnD-like phenotype may further contribute to persistent microglial activation. Black arrows indicate pathological progression, blue arrows indicate feedback loops, and bidirectional arrows indicate mutual reinforcement. Solid lines represent established or relatively well-supported mechanisms, whereas dashed lines represent proposed, indirect, or context-dependent interactions. Aβ, myloid-β; AGEs, dvanced glycation end products; ATP, denosine triphosphate; DAMPs, amage-associated molecular patterns; DAM, isease-associated microglia; FFAs, ree fatty acids; HMGB1, igh mobility group box 1; IL-1β, nterleukin-1β; IL-18, nterleukin-18; MGnD, icroglial neurodegenerative phenotype; NF-κB, uclear factor-κB; NLRP3, NLR family pyrin domain-containing 3; PRRs, attern-recognition receptors; ROS, eactive oxygen species.

### 4.5 Complement-mediated synaptic pruning and microglia-driven synaptic injury

Synaptic damage is a major determinant of early cognitive and neurological dysfunction in neurodegenerative diseases and often precedes overt neuronal death (Tzioras et al., 2023; Ye et al., 2026). In the context of BIR, synapses themselves become more metabolically vulnerable, while abnormalities in the inflammatory and phagocytic functions of microglia gradually transform normal synaptic maintenance and pruning into pathological injury (Sun and Mi, 2025). This section therefore focuses on how the immunometabolic imbalance and inflammatory activation of microglia described above contribute to structural and functional synaptic abnormalities.

Complement-mediated synaptic pruning is a key mechanism by which microglia maintain synaptic homeostasis. Under physiological conditions, the C1q, C3, and CR3 pathway is involved in the recognition and clearance of redundant or dysfunctional synapses during development (Ge et al., 2024; Andoh et al., 2025; Hong et al., 2016). However, under chronic metabolic and inflammatory stress, complement-dependent synaptic recognition and pruning may become dysregulated. Current evidence supports complement activation as an important contributor to aberrant synaptic elimination in neurodegenerative models, but the direct causal link between BIR and complement-dependent synaptic pruning remains incompletely established. Thus, complement activation should be considered one component of a broader network of synaptic recognition, phagocytic, and inflammatory pathways rather than the sole mechanism responsible for synaptic injury.

Complement-independent or pro-resolving pathways may also provide protective alternatives. Phosphatidylserine recognition through TAM receptors and their ligands Gas6 and Protein S can facilitate the clearance of cellular debris and synaptic material, while potentially supporting resolution of inflammatory responses. In addition, specialized pro-resolving lipid mediators such as protectin D1 have been reported to promote neuronal survival and enhance Aβ42 phagocytosis in experimental systems. These pathways therefore represent candidate approaches for restoring balanced phagocytic and resolution functions. However, their therapeutic roles in BIR-associated neurodegeneration remain experimental and have not been established in clinical studies (Beiter et al., 2024; Zhu et al., 2016).

In addition to phagocytosis, inflammatory mediators can directly impair synaptic function. NLRP3-associated inflammatory mediators, including IL-1β and TNF-α, can impair synaptic plasticity, including hippocampal long-term potentiation (Lopez-Rodriguez et al., 2021). At the same time, microglia-derived reactive oxygen species and nitric oxide can damage synaptic membrane lipids and local mitochondria, thereby further reducing synaptic energy supply and structural stability (Picca et al., 2020; Du et al., 2026). Thus, inflammatory mediators and oxidative stress act synergistically on synapses that are already metabolically fragile, increasing their susceptibility to dysfunction.

In neurodegenerative diseases such as AD and PD, β amyloid oligomers and aggregated α-synuclein can further aggravate synaptic injury through complement dependent mechanisms and microglia-driven inflammation (Zhang et al., 2024; Lv et al., 2024; Frigerio et al., 2025; Huang et al., 2023). Meanwhile, microglial lipid-handling disturbances that may accompany BIR—including altered lipid uptake through scavenger receptors such as CD36, impaired fatty-acid utilization, cholesterol and lipoprotein trafficking, lipid-droplet accumulation, and lysosomal lipid processing—may alter the composition and function of extracellular vesicles released by microglia (Loving and Bruce, 2020; Guo et al., 2025; Guo et al., 2020), changing the lipids, microRNAs, and pathological proteins they carry and thereby influencing neuronal function. However, compared with complement mediated pruning and cytokine driven synaptic dysfunction, direct evidence for extracellular vesicle mediated long range synaptic damage remains limited.

Thus, microglia-mediated synaptic injury is not simply the consequence of increased phagocytic activity. Rather, it reflects the combined effects of increased synaptic vulnerability, complement mediated misrecognition, sustained inflammatory signaling, and local oxidative damage. In this way, upstream metabolic abnormalities and inflammatory responses are translated into structural and functional deficits at the neural network level, making synaptic damage a key intermediate step in BIR-associated neurodegeneration. Selective complement modulation may therefore be preferable to broad complement suppression, given the physiological roles of complement in synaptic maintenance and tissue homeostasis (Tenner and Petrisko, 2025).

### 4.6 Feed-forward loops between brain insulin resistance and microglial inflammation

Brain insulin resistance and microglia-mediated neuroinflammation are not independent pathological processes. Rather, they may form a self sustaining pathogenic loop through reciprocal reinforcement (Sun and Mi, 2025; Arnold et al., 2018). The preceding sections have described this interaction from the perspectives of abnormal insulin signaling, disrupted organelle homeostasis, NLRP3 inflammasome activation, and synaptic injury. However, these mechanisms do not operate in isolation, but remain interconnected throughout disease progression.

On the one hand, BIR impairs the capacity of microglia to maintain immunometabolic homeostasis, thereby lowering their threshold for inflammatory activation (Shah and Doshi, 2026). Impaired Akt signaling and the resulting disturbances in mitochondrial, redox, and lipid lysosomal homeostasis render microglia more sensitive to pathological stimuli and promote sustained activation of inflammatory pathways such as NF-κB and NLRP3. Therefore, BIR not only increases the likelihood of an inflammatory response but may also reduce the ability of microglia to return to homeostasis once activated.

On the other hand, persistent microglial inflammation can further exacerbate defects in insulin signaling (Lo and Zeng, 2025). Inflammatory mediators such as TNF-α, IL-1β, IL-6, reactive oxygen species, and nitric oxide can impair insulin signaling in neurons and glial cells and, through stress-related pathways including JNK, IKKβ, PKC, and mTOR with S6K, promote abnormal IRS serine phosphorylation, thereby further inhibiting PI3K and Akt signaling (Hotamisligil, 2010). Persistent inflammation and oxidative stress may also damage the blood–brain barrier and neurovascular units, rendering the central microenvironment more susceptible to peripheral inflammatory and metabolic stressors (Shah and Doshi, 2026; Lo and Zeng, 2025).

As neuronal and synaptic injury progresses, damage associated molecular patterns such as ATP, HMGB1, mitochondrial DNA, lipid peroxides, and abnormal protein aggregates continue to accumulate and further stimulate microglia. Thus, neuronal injury is not only a consequence of chronic inflammation but also an important driver of sustained microglial activation. As a result, the initial metabolic disturbances associated with BIR may gradually evolve into a pathological state maintained by microglial inflammation, neuronal injury, and further impairment of insulin signaling.

It is important to emphasize that this bidirectional feedback model does not imply that BIR is the initiating event in all neurodegenerative diseases. Current evidence is derived mainly from animal models, *in vitro* experiments, and cross sectional studies of the human brain, making it difficult to establish the temporal sequence between BIR and microglial inflammation across different diseases and disease stages.

Future research should integrate cell-type-specific regulation of insulin signaling, microglia-targeted interventions, and longitudinal analyses to clarify the causal contributions of distinct mechanistic nodes to disease onset and progression. In parallel, longitudinal population based studies and multimodal biomarker analyses will help define the temporal relationships among BIR, microglial activation, and neurodegenerative pathology, thereby further assessing the therapeutic value of interrupting this feedback loop.

In summary, BIR, microglial immunometabolic imbalance, chronic neuroinflammation, and neurodegenerative injury may form a mutually reinforcing pathological network, the principal mechanisms and potential feedback relationships of which are summarized in Figure 2.

**Figure 2:**
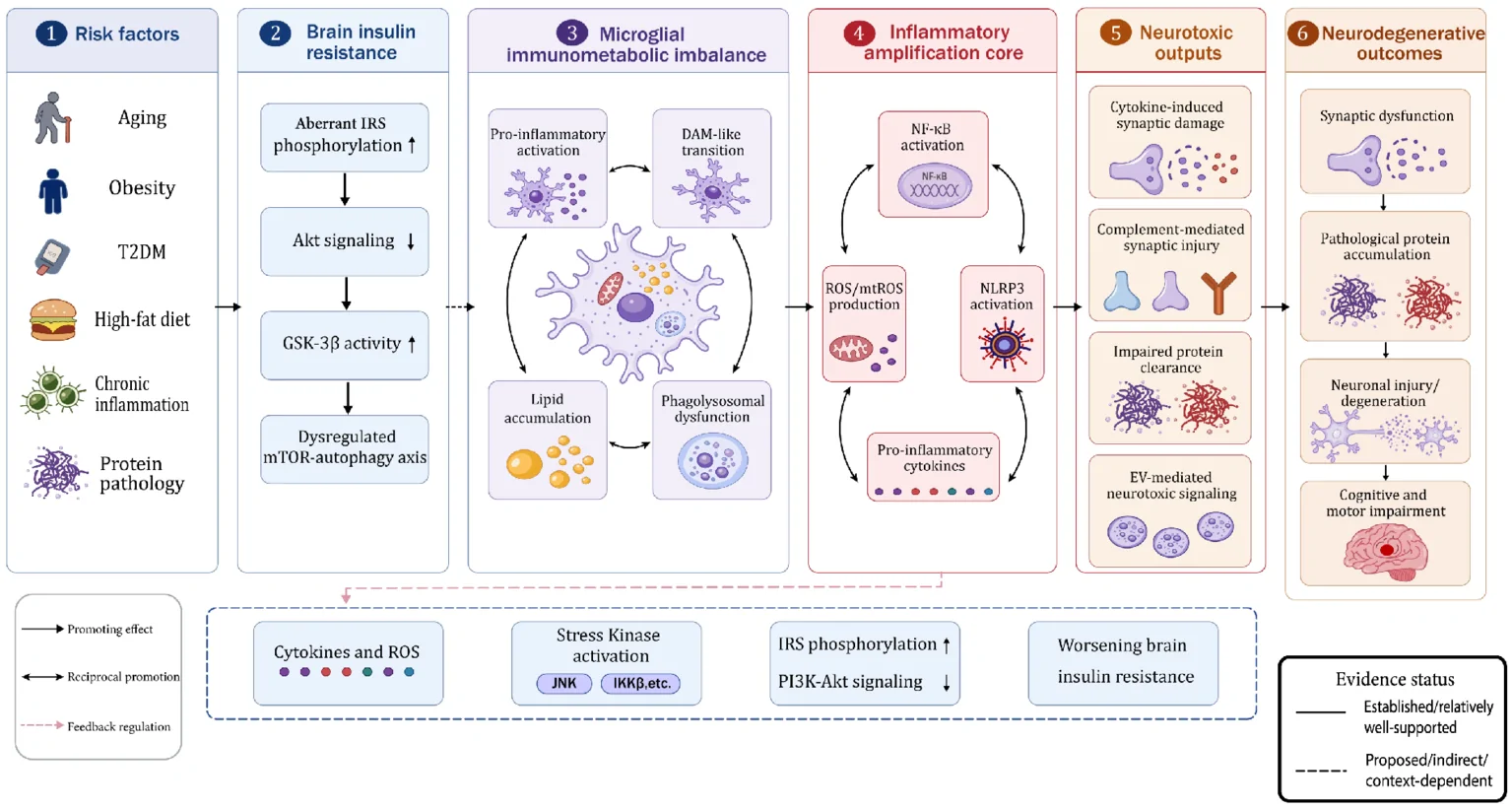
Overview of the proposed mechanism linking brain insulin resistance to neurodegeneration. Metabolic and inflammatory risk factors may contribute to brain insulin resistance, which is associated with impaired insulin signaling and microglial immunometabolic imbalance, including pro-inflammatory activation, DAM-like transition, lipid accumulation, and phagolysosomal dysfunction. These alterations converge on an inflammatory amplification core involving NF-κB, ROS/mtROS, NLRP3 inflammasome activation, and pro-inflammatory cytokines, ultimately contributing to synaptic injury, impaired protein clearance, neuronal degeneration, and cognitive and motor impairment. Cytokines, ROS, stress kinase activation, impaired insulin signaling, and worsening brain insulin resistance may form reciprocal feedback mechanisms that sustain chronic neuroinflammation. Solid lines represent established or relatively well-supported mechanisms, whereas dashed lines represent proposed, indirect, or context-dependent interactions. Akt, protein kinase B; DAM, disease-associated microglia; EV, extracellular vesicle; GSK-3β, glycogen synthase kinase-3β; IRS, insulin receptor substrate; mtROS, mitochondrial reactive oxygen species; mTOR, mechanistic target of rapamycin; NF-κB, nuclear factor-κB; NLRP3, NLR family pyrin domain-containing 3; ROS, reactive oxygen species; T2DM, type 2 diabetes mellitus.

## 5 Disease-specific evidence in neurodegenerative disorders

### 5.1 Alzheimer disease: interactions among brain insulin resistance, proteinopathy, and microglial dysfunction

AD is the most common neurodegenerative dementia and is characterized by progressive cognitive decline, Aβ accumulation, tau hyperphosphorylation, synaptic loss, and neuronal degeneration. Increasing evidence indicates that AD involves not only abnormal protein aggregation but also cerebral metabolic dysfunction and persistent neuroinflammation (Sun and Mi, 2025; Barone and Butterfield, 2025). Within this pathological network, BIR may provide an important metabolic context in which abnormal protein homeostasis and microglial dysfunction become mutually reinforcing.

Among neurodegenerative diseases, AD has been most extensively investigated in relation to BIR (Tramutola et al., 2020; Arvanitakis et al., 2020). Reduced insulin receptor expression, abnormal IRS phosphorylation, and attenuated PI3K/Akt signaling have been reported in the brains of patients with AD, suggesting impaired central insulin responsiveness (Leclerc et al., 2023). The concept of “type 3 diabetes” has therefore been proposed to emphasize the potential contribution of impaired brain insulin signaling to AD, although this terminology remains controversial (Kciuk et al., 2024). Current evidence more appropriately supports the view that BIR represents a metabolic abnormality within the pathological network of AD rather than a distinct disease entity (Biswas et al., 2025).

A major disease-specific interface between BIR and AD involves Aβ metabolism. Impaired insulin signaling may affect Aβ synthesis, degradation, and transport, thereby reducing Aβ clearance and promoting its accumulation in the brain. Conversely, Aβ oligomers can interfere with insulin receptor function and downstream signaling, further impairing insulin responsiveness in neurons and glial cells (Wakabayashi et al., 2019; Kellar and Craft, 2020). Thus, BIR and Aβ pathology may reinforce each other through a bidirectional relationship. In parallel, impaired insulin signaling may influence tau homeostasis through reduced Akt activity and weakened inhibitory control of GSK3β, thereby promoting pathological tau phosphorylation (Stanley et al., 2016; Ng et al., 2016). These interactions suggest that BIR may contribute to AD progression by affecting both major proteinopathic pathways rather than acting solely through inflammatory mechanisms.

Microglia provide an additional interface between metabolic dysfunction and AD proteinopathy. During the early stages of Aβ accumulation, microglia can recognize and phagocytose Aβ and thereby contribute to the clearance of abnormal proteins. However, persistent exposure to Aβ and the progressive deterioration of the pathological microenvironment may impair microglial phagocytic and lysosomal processing capacity, while increasing the production of pro-inflammatory mediators (Keren-Shaul et al., 2017; Marschallinger et al., 2020; Gonçalves et al., 2019). This functional transition may shift microglia from an initially protective clearance state toward a state that contributes to persistent inflammatory injury. Importantly, the resulting dysfunction is not limited to cytokine release; impaired phagolysosomal function may also compromise the continued clearance of pathological proteins, thereby further increasing the pathological burden.

The consequences of this interaction are particularly evident at the synaptic level. BIR-associated metabolic dysfunction and impaired synaptic plasticity may increase neuronal susceptibility to Aβ and inflammatory stress (de la Monte, 2017). At the same time, Aβ oligomers and the inflammatory microenvironment can promote complement-mediated synaptic tagging and enhance aberrant microglial engulfment of synapses. Thus, microglial dysfunction in AD may contribute to cognitive decline through two interconnected processes: impaired clearance of pathological proteins and excessive removal of functional synapses.

Overall, AD provides a disease context in which BIR is closely interconnected with proteinopathy and microglial dysfunction. Rather than representing a simple pathway from BIR to AD, the available evidence supports a bidirectional pathological network involving impaired insulin signaling, Aβ and tau abnormalities, altered microglial clearance, and synaptic injury. Although these interactions are increasingly supported, their causal relationships remain incompletely resolved, and some mechanistic evidence is derived primarily from animal models or indirect studies. Further cell type-specific and longitudinal investigations are therefore required to determine the temporal and causal relationships among BIR, microglial dysfunction, and AD progression.

### 5.2 Parkinson disease: interactions among brain insulin resistance, α-synuclein, and mitochondrial vulnerability

PD is the second most common neurodegenerative disease and is characterized pathologically by progressive degeneration of dopaminergic neurons in the substantia nigra pars compacta and accumulation of α-synuclein in Lewy bodies (Surmeier et al., 2017). In addition to abnormal protein aggregation, PD involves metabolic abnormalities, mitochondrial dysfunction, and immune-inflammatory alterations (Roodveldt et al., 2024; Li et al., 2025; Leandrou et al., 2024). In this context, the relationship between BIR and PD may be particularly relevant to the metabolic vulnerability of nigral neurons and the immune responses triggered by α-synuclein pathology.

Clinical and experimental studies suggest that impaired insulin signaling may occur in the brains of patients with PD, including altered IRS phosphorylation, reduced Akt activity, and increased GSK3β activity (Bassil et al., 2017; Tansey et al., 2022). Epidemiological evidence further indicates that diabetes and peripheral insulin resistance may increase the risk of PD and may be associated with faster disease progression (Nowell et al., 2023; Rhee et al., 2020). These observations support an association between impaired insulin metabolism and PD, although they do not by themselves establish a direct causal relationship. Within the brain, BIR may reduce the metabolic and organelle resilience of both neurons and microglia, thereby increasing their susceptibility to disease-associated stress.

A major disease-specific interaction involves α-synuclein and microglia. Aggregated α-synuclein can activate microglia and induce inflammatory mediator production and oxidative stress responses (Bassil et al., 2017; Rhee et al., 2020; Wong and Krainc, 2017; Bido et al., 2021). Conversely, activated microglia may contribute to the propagation and persistence of α-synuclein-associated pathology, creating a reciprocal relationship between protein aggregation and immune activation. This interaction provides a disease-specific context in which the metabolic and inflammatory vulnerabilities described in Section 4 may be engaged by α-synuclein pathology. Rather than viewing microglial activation as an isolated downstream event, it may therefore be more appropriate to consider α-synuclein and microglia as components of a self-reinforcing pathological network.

PD exhibits a disease-specific vulnerability to mitochondrial and oxidative stress in nigral dopaminergic neurons, which may interact with the broader mitochondrial and immunometabolic disturbances discussed in Section 4.3. BIR may therefore increase the metabolic vulnerability of both dopaminergic neurons and microglia, while α-synuclein pathology provides an additional disease-specific inflammatory stimulus.

Thus, compared with AD, in which BIR is closely intertwined with Aβ and tau pathology, PD may represent a disease context in which BIR–microglial dysfunction converges more strongly with α-synuclein pathology and the mitochondrial vulnerability of nigral dopaminergic neurons. BIR may not act as an independent initiating factor but may instead reduce the stress resilience of both neurons and microglia, thereby facilitating the persistence of α-synuclein-associated immune activation and neuronal degeneration.

Taken together, AD and PD share several metabolic and inflammatory features, including impaired insulin signaling, mitochondrial dysfunction, oxidative stress, chronic microglial activation, and impaired proteostasis. However, the disease-specific interfaces differ. In AD, BIR is particularly closely linked to Aβ and tau metabolism, synaptic dysfunction, and microglial Aβ clearance. In PD, the relationship between BIR and α-synuclein pathology is accompanied by a distinctive vulnerability of nigrostriatal dopaminergic neurons to oxidative stress, dopamine metabolism, iron accumulation, and mitochondrial dysfunction. Thus, the BIR–microglia axis may represent a shared upstream or amplifying mechanism, whereas the downstream pathological consequences are shaped by disease-specific proteinopathies and neuronal vulnerabilities. These similarities should therefore not be interpreted as evidence that AD and PD share an identical pathogenic mechanism (Xiang et al., 2026).

### 5.3 Implications for other neurodegenerative conditions

Beyond AD and PD, BIR and microglial dysfunction may also participate in other neurodegenerative disorders, including dementia with Lewy bodies (DLB), amyotrophic lateral sclerosis (ALS), and Huntington’s disease (McDonald et al., 2023; Gao et al., 2023). DLB shares α-synuclein pathology with PD and is associated with altered microglial responses; however, direct evidence linking BIR specifically to microglial dysfunction in DLB remains limited. Available evidence therefore supports mechanistic overlap with PD rather than a disease-specific BIR–microglia pathway (Gao et al., 2023). In ALS, microglial responses exhibit substantial stage dependence, with more pronounced pro-inflammatory and potentially neurotoxic characteristics during advanced disease stages. Although insulin resistance and metabolic abnormalities have been reported in ALS, direct evidence connecting brain insulin resistance to microglial insulin signaling remains limited. The proposed BIR–microglia relationship should therefore be regarded as preliminary rather than established (Chen et al., 2024; Gao et al., 2023; Hlushchuk et al., 2022).

Huntington’s disease (HD) may also involve impaired insulin signaling and microglial immunometabolic dysfunction. Experimental studies suggest that mutant huntingtin can impair neuronal insulin signaling, while recent evidence indicates that altered microglial metabolism and pro-inflammatory activation may contribute to HD pathology (Chang et al., 2018; Jauhari et al., 2026). However, direct evidence establishing a BIR-driven microglial mechanism remains limited, and current support is primarily experimental.

Overall, direct evidence linking BIR to these disorders remains considerably more limited than that available for AD and PD. The available findings therefore support the possibility that altered insulin signaling and microglial immunometabolism represent a broader pathological mechanism, but disease-specific differences should be carefully considered. In particular, mechanisms established in AD or PD should not be extrapolated uncritically to other neurodegenerative diseases. Future studies should determine whether BIR produces comparable microglial metabolic changes across different disease contexts and how these changes interact with disease-specific pathological proteins, neuronal vulnerabilities, and disease stages.

## 6 Therapeutic strategies targeting the BIR–microglia–inflammation axis

The therapeutic evidence discussed below spans different translational stages and should therefore be interpreted accordingly. Intranasal insulin and GLP-1 receptor agonists have undergone clinical investigation, whereas many microglia-directed interventions, including NLRP3 inhibition, complement modulation, and metabolic reprogramming strategies, remain predominantly preclinical. Accordingly, experimental efficacy should not be equated with established clinical benefit.

### 6.1 Restoring brain insulin signaling

Improving brain insulin signaling is a major strategy for intervening in BIR-associated neurodegenerative diseases (Kellar and Craft, 2020; Gao et al., 2023). Intranasal insulin is a representative approach for enhancing central insulin action (Craft et al., 2020; Hallschmid, 2021). Unlike peripheral administration, intranasal delivery can partially bypass the blood–brain barrier, allowing insulin to enter the central nervous system and act across multiple brain regions (Craft et al., 2020; Wingrove et al., 2019). Clinical studies have shown that intranasal insulin may improve memory performance in some patients with mild cognitive impairment and early AD (Craft et al., 2020; Ferrari et al., 2022), and has shown preliminary to improve motor function and verbal fluency in patients with PD (Novak et al., 2019). However, larger and longer-term randomized controlled trials have not consistently demonstrated clinical benefit, and a phase 2/3 randomized clinical trial did not show cognitive or functional benefits over 12 months (Craft et al., 2020). Overall, clinical evidence remains mixed, and disease-modifying efficacy has not been established.

Metformin exerts insulin sensitizing effects primarily through regulation of energy metabolism pathways such as AMPK and may also influence the immunometabolic state of microglia (Cimini et al., 2026; González Pérez et al., 2025). Existing studies suggest that its potential neuroprotective effects may be related not only to improved metabolic homeostasis but also to suppression of pro-inflammatory signaling and modulation of microglial energy utilization (Cimini et al., 2026; González Pérez et al., 2025; Rena et al., 2017). However, the clinical efficacy of metformin in neurodegenerative diseases has not yet been consistently demonstrated, and its effects may vary depending on dosage, disease stage, peripheral glucose metabolism, and central drug exposure (Cimini et al., 2026). Thus, current evidence remains predominantly preclinical or observational, and metformin cannot currently be considered an established disease-modifying therapy for neurodegenerative disease.

Glucagon-like peptide-1 (GLP-1) receptor agonists have attracted increasing attention as potential therapies that may simultaneously target metabolic dysfunction and neuroinflammation. GLP-1 signaling is present in the brain and may influence neuronal and metabolic functions through multiple signaling pathways (Rosen and Ingelfinger, 2026; Montaner et al., 2024; Cork et al., 2015). Preclinical studies and clinical trials have suggested potential benefits of GLP-1 receptor agonists in neurodegenerative disorders, including effects on cognitive or motor function and inflammatory responses. In PD, clinical evidence remains mixed. Although earlier phase 2 studies suggested potential benefits, the phase 3 exenatide trial did not demonstrate a disease-modifying benefit compared with placebo, indicating that the translational efficacy of GLP-1 receptor agonism remains uncertain (Vijiaratnam et al., 2025). Because microglia–astrocyte interactions can contribute to neuroinflammatory amplification in PD, modulation of these cellular responses may represent one possible mechanism underlying the neuroprotective effects of GLP-1-related therapies (Yun et al., 2018).

### 6.2 Anti-inflammatory and immunometabolic therapies targeting microglia

Given the dual protective and detrimental roles of microglia in BIR-associated neurodegeneration, therapeutic strategies should aim to restore appropriate microglial states rather than simply suppress microglial activation (Bernier et al., 2020). NLRP3 inflammasome inhibitors are currently a major focus of investigation. Inhibition of NLRP3 can reduce caspase-1 activation and IL-1β release, thereby attenuating microglial inflammation and pyroptosis. For example, the small molecule inhibitor MCC950 suppresses NLRP3 inflammasome activation and alleviates pro-inflammatory responses and synaptic dysfunction in multiple neuroinflammatory models (Coll et al., 2015). However, its safety, pharmacokinetic profile, and clinical efficacy still require further evaluation. Thus, NLRP3 inhibition remains predominantly preclinical and should not yet be considered an established clinical therapy.

Modulation of microglial metabolism represents another promising strategy (Bernier et al., 2020). Targeting pathways such as AMPK, mTOR, and mitochondrial quality control may improve aberrant glycolysis, mitochondrial dysfunction, and oxidative stress, thereby reducing persistent inflammatory activation (Pickrell and Youle, 2015; Bernier et al., 2020). Similarly, interventions directed at lipid metabolism and lysosomal function may enhance the processing of pathological lipids and cellular debris. TREM2, a receptor closely associated with microglial phagocytosis and lipid metabolism, may help preserve microglial adaptability to lipid burden through its downstream signaling pathways (Deczkowska et al., 2020). Compared with simply suppressing inflammatory mediators, simultaneous restoration of energy metabolism and cellular clearance capacity may therefore better align with the pathological characteristics of BIR-related chronic neuroinflammation. Evidence for these approaches remains predominantly preclinical, with limited clinical validation in neurodegenerative disease.

Overall, therapeutic strategies targeting microglia are shifting from simple suppression of downstream inflammatory pathways toward broader regulation of immune metabolism and cellular homeostasis. Because microglial functions are highly state dependent and stage dependent, future interventions will need to suppress pathological inflammation while preserving the normal phagocytic, clearance, and trophic support functions of microglia, and will also need to define the optimal timing of intervention across disease stages.

### 6.3 Emerging therapeutic perspectives

Beyond insulin sensitization and direct microglia-targeted approaches, immunometabolic strategies, the gut-brain axis, and novel delivery platforms also offer potential therapeutic directions for BIR-related neurodegenerative disorders. Immunometabolic interventions may improve microglial function by modulating glycolysis, NAD + metabolism, mitophagy, and lipid metabolism (Bernier et al., 2020; Zhao et al., 2024; Miao et al., 2023). The gut microbiota and its metabolites can influence metabolic homeostasis and microglial states through the gut–brain axis. Thus, interventions targeting the microbiota or its metabolites may represent a potential therapeutic direction for BIR-associated neurodegenerative disorders (Cryan et al., 2019; Erny et al., 2015). In addition, exosome-based and microRNA delivery approaches have emerged as potential strategies for modulating neuroinflammation and neural repair (Losurdo et al., 2020; Xin et al., 2013). However, important limitations remain with respect to targeting specificity, dose control, safety, and manufacturing standardization.

Dual GIP and GLP-1 receptor agonists have also attracted increasing attention. Preclinical studies suggest that combined GLP-1/GIP receptor activation may exert neuroprotective effects by improving insulin sensitivity and modulating inflammatory and mitochondrial pathways. Animal studies have reported beneficial effects in AD and PD models (Zhou et al., 2025; Athauda et al., 2026; Hölscher, 2018), but their clinical value remains to be established.

In summary, BIR and microglial dysfunction may contribute to the onset and progression of multiple neurodegenerative diseases through diverse mechanisms, and therapeutic strategies targeting insulin signaling, microglial function, and related metabolic abnormalities are receiving growing attention. Table 1 summarizes BIR-related changes, microglial responses, neuronal consequences, and potential therapeutic approaches across different neurodegenerative disorders.

**Table 1:** Summary of brain insulin resistance, microglial responses, neuronal consequences, and potential therapeutic directions across neurodegenerative disorders.

Disease	Key BIR-related alteration	Major microglial response	Principal neuronal consequence	Potential therapeutic directions	Evidence strength
AD	Impaired IRS/PI3K/Akt signaling; increased GSK3β activity; impaired Aβ clearance	Chronic activation; impaired phagocytosis; NLRP3 activation; complement-dependent synaptic engulfment	Aβ/tau accumulation, synaptic loss, cognitive decline	Intranasal insulin, GLP-1R agonists, NLRP3 inhibitors, TREM2-targeted approaches	Relatively strong; supported by human and experimental evidence, although direct microglial evidence is still emerging
PD	Impaired insulin signaling; reduced Akt activity; mitochondrial dysfunction and oxidative stress	α-syn-induced activation; TLR2/TLR4-mediated inflammatory responses; impaired α-syn clearance	Dopaminergic neuronal degeneration; α-syn propagation; motor dysfunction	GLP-1 receptor agonists; immunometabolic and mitochondrial-targeted strategies; NLRP3 inhibition	Moderate; supported by experimental and clinical/epidemiological evidence, but direct microglial BIR evidence remains limited
DLB	Potential impairment of insulin signaling and metabolic homeostasis	Chronic microglial activation associated with α-syn pathology	Cortical and subcortical neurodegeneration; cognitive decline	Insulin-sensitizing and anti-inflammatory strategies requiring further validation	Limited; evidence for microglial involvement exists, but direct BIR–microglial evidence is sparse
ALS	Metabolic dysregulation and potential impairment of insulin signaling	Stage-dependent microglial phenotypic changes; increased pro-inflammatory and neurotoxic responses in later stages	Motor neuron metabolic stress and degeneration; progressive motor dysfunction	Immunometabolic regulation and mitochondrial-targeted strategies requiring further validation	Limited; metabolic and microglial abnormalities are reported, but a direct BIR–microglial causal link remains insufficiently established
HD	Impaired insulin signaling and metabolic dysfunction associated with disease progression	Microglial activation; pro-inflammatory responses and altered immunometabolic state	Striatal and cortical neuronal degeneration; progressive cognitive and motor dysfunction	Insulin-sensitizing, metabolic, and anti-inflammatory strategies requiring further validation	Limited/emerging; experimental evidence supports metabolic and microglial involvement, but direct BIR-specific evidence remains preliminary

BIR, brain insulin resistance; IRS, insulin receptor substrate; PI3K, phosphoinositide 3-kinase; Akt, protein kinase B; TLR, Toll-like receptor; NLRP3, NLR family pyrin domain-containing 3 inflammasome; Aβ, amyloid-β; α-syn, α-synuclein; AD, Alzheimer disease; PD, Parkinson disease; DLB, dementia with Lewy bodies; ALS, amyotrophic lateral sclerosis; HD, Huntington’s disease; GLP-1R, glucagon-like peptide-1 receptor. Evidence strength reflects the relative convergence of human, experimental, and cell-type-specific evidence linking BIR and microglial dysfunction in each disease; it does not represent a formal evidence-grading system.

## 7 Discussion: current limitations and future directions

Although increasing evidence supports a close relationship among brain insulin resistance, microglial dysfunction, chronic neuroinflammation, and neurodegenerative disease, several key limitations remain. Addressing these issues will be important for clarifying the mechanistic role of the axis linking brain insulin resistance, microglia, and inflammation, and for promoting its translational application.

### 7.1 Distinguishing central and peripheral insulin resistance

One major challenge is that the relationship between peripheral insulin resistance and brain insulin resistance remains difficult to define precisely. Peripheral metabolic disorders can affect the central nervous system through systemic inflammation, vascular dysfunction, altered blood–brain barrier permeability, and changes in circulating insulin, glucose, and lipid levels. These peripheral abnormalities may indirectly influence neuronal activity, glial responses, and cerebral metabolism.

However, brain insulin resistance may also arise locally within the central nervous system, even in the absence of overt peripheral metabolic disease. Neurons, astrocytes, endothelial cells, and microglia may each develop impairments in insulin signaling under conditions of aging, oxidative stress, protein aggregation, or chronic inflammation. Therefore, future studies should more clearly distinguish central from peripheral insulin resistance and determine how systemic metabolic disturbances interact with local insulin signaling abnormalities in the brain.

A better distinction between peripheral and central insulin resistance will also be important for clinical translation. Biomarkers that reflect systemic insulin resistance may not fully represent insulin signaling status in the brain. Future studies should therefore combine peripheral metabolic indicators with cerebrospinal fluid markers, neuroimaging, and molecular signatures related to specific cell types to more accurately evaluate brain insulin resistance in neurodegenerative diseases.

### 7.2 Defining insulin signaling in microglia at the cell type level

Another important limitation is the still limited cell-specific evidence regarding insulin signaling dysfunction in human microglia. Direct experimental evidence is now emerging: microglia-specific insulin receptor deletion in mice alters microglial metabolism and innate immune programs, impairs Aβ uptake, and exacerbates AD-like pathology (Chen et al., 2025). However, most detailed molecular mechanisms involving IRS phosphorylation, stress kinases, and feedback between inflammatory signaling and insulin resistance remain derived from peripheral metabolic tissues, neuronal or astrocytic models, or non-cell-specific experimental systems. These findings provide biologically plausible mechanisms but should not be interpreted as definitive evidence that the same molecular events occur in human microglia. Future studies using human iPSC-derived microglia, spatial transcriptomics, cell-specific phosphoproteomics, and *in vivo* metabolic tracing will be important for establishing direct causal relationships.

Future studies should therefore focus on insulin signaling mechanisms in microglia. Microglia specific genetic manipulation, conditional knockout models, lineage tracing, single cell sequencing, spatial transcriptomics, and proteomics with resolution at the cell type level may help clarify whether impaired insulin signaling directly reprograms microglial immunometabolism. These approaches may also help determine whether brain insulin resistance promotes microglial inflammatory priming through intrinsic changes within microglia or indirectly through neuronal injury, astrocyte activation, vascular dysfunction, or altered extracellular metabolites.

In addition, future work should define the specific downstream pathways by which insulin signaling regulates microglial function. These may include IRS/PI3K/Akt signaling, GSK3β activity, mTOR dependent metabolic regulation, FOXO mediated stress responses, Nrf2 related antioxidant defense, mitochondrial homeostasis, autophagy, lysosomal degradation, and lipid metabolism. Clarifying these mechanisms will be essential for determining whether microglial insulin signaling represents a direct therapeutic target.

### 7.3 Establishing temporal and causal relationships

The temporal and causal relationships among brain insulin resistance, microglial activation, and neurodegeneration remain unresolved. In many neurodegenerative diseases, impaired insulin signaling, mitochondrial dysfunction, oxidative stress, abnormal protein aggregation, synaptic damage, and neuroinflammation occur simultaneously and reinforce one another. Therefore, it remains difficult to determine whether brain insulin resistance is an initiating event, a disease amplifier, or a consequence of chronic neurodegenerative pathology.

Longitudinal animal models and intervention studies designed according to disease stage are needed to clarify when brain insulin resistance emerges during disease progression. For example, it remains unclear whether impaired insulin signaling precedes microglial inflammatory priming, or whether chronic microglial activation and cytokine release gradually disrupt insulin signaling in neurons and glial cells. These two possibilities are not mutually exclusive and may form a bidirectional pathological cycle.

Future clinical studies should also adopt longitudinal designs to determine whether brain insulin resistance predicts cognitive decline, motor impairment, biomarker progression, or neuroimaging changes. Such studies may help identify disease stages during which restoring insulin signaling or modulating microglial inflammation would be most effective. This is particularly important because therapeutic interventions targeting inflammation or metabolism may have different effects at early, intermediate, and late stages of neurodegenerative disease.

### 7.4 Integrating microglial heterogeneity, multi omics, imaging, and translational models

Microglial heterogeneity represents another major challenge. Microglia do not exist in a single activated state, but instead display diverse context dependent phenotypes shaped by aging, sex, brain region, disease stage, local metabolic stress, and interactions with neurons, astrocytes, oligodendrocytes, and vascular cells. Therefore, the influence of brain insulin resistance on microglia may vary across different diseases, anatomical regions, and pathological stages.

Future studies should move beyond simplified inflammatory and inflammation resolving classifications. Instead, it will be important to define how brain insulin resistance shapes specific microglial subpopulations, including disease-associated, lipid associated, interferon responsive, senescent like, and metabolically stressed microglial states. Single cell RNA sequencing, spatial transcriptomics, proteomics, metabolomics, and lipidomics may help identify microglial signatures that are specifically associated with insulin resistant brain environments.

In parallel, advanced neuroimaging and biomarker studies are needed to improve translational relevance. Positron emission tomography, magnetic resonance imaging, cerebrospinal fluid biomarkers, blood based biomarkers, and longitudinal cognitive or behavioral assessments may help connect molecular mechanisms observed in experimental models with disease progression in humans. Combining these approaches may also support patient stratification and facilitate the development of interventions tailored to disease stage.

Finally, therapeutic strategies targeting the axis linking brain insulin resistance, microglia, and inflammation require further validation. Intranasal insulin, insulin sensitizers, GLP-1 receptor agonists, inflammation suppressing strategies, NLRP3 inflammasome inhibitors, mitochondrial modulators, and interventions enhancing autophagy have shown potential in experimental or clinical contexts. However, their efficacy may depend on disease stage, metabolic status, genetic background, sex, age, and the extent of irreversible neuronal loss. Therefore, future therapies should aim not simply to suppress microglial activation, but to restore microglial homeostasis, metabolic flexibility, phagocytic capacity, and protective immune functions.

The feasibility of these strategies should be evaluated against the current stage of therapeutic development. Approaches that restore microglial homeostasis or enhance pathological protein clearance are supported primarily by mechanistic and preclinical studies, whereas interventions such as intranasal insulin and GLP-1 receptor agonists have entered clinical testing but have produced heterogeneous results. For example, the phase 3 exenatide trial in Parkinson’s disease did not demonstrate a disease-modifying benefit, highlighting the gap between promising preclinical mechanisms and clinical efficacy (Vijiaratnam et al., 2025). Similarly, complement-targeted strategies remain largely preclinical and require careful consideration of target specificity and treatment timing because complement also performs physiological synaptic and tissue-maintenance functions (Tenner and Petrisko, 2025). Therefore, future therapeutic strategies should be prioritized according to target specificity, central nervous system exposure, disease stage, and the reversibility of the underlying pathology rather than assuming that suppression of a single inflammatory pathway will be sufficient.

## 8 Conclusion

Brain insulin resistance is increasingly recognized as more than a disturbance of neuronal glucose metabolism. It represents a broader neuroimmunometabolic disorder that can reshape the central nervous system microenvironment and promote chronic neuroinflammation. In this process, microglia are key cellular mediators linking impaired insulin signaling to inflammatory amplification, mitochondrial dysfunction, oxidative stress, defective autophagy and lysosomal activity, lipid metabolic stress, NLRP3 inflammasome activation, complement mediated synaptic injury, and progressive neuronal damage.

The evidence summarized in this review suggests that brain insulin resistance and inflammation mediated by microglia may form a pathological cycle that reinforces itself. Impaired insulin signaling lowers the threshold for microglial inflammatory activation, while persistent microglia-derived cytokines, oxidative stress, and damage associated signals further disrupt insulin signaling in neurons and glial cells. This vicious cycle may contribute to the initiation and progression of neurodegenerative diseases such as AD and PD.

Targeting the axis linking brain insulin resistance, microglia, and inflammation may therefore provide a promising therapeutic direction. Rather than focusing only on neuronal metabolism or nonspecific suppression of inflammation, future strategies should aim to restore insulin signaling, preserve microglial homeostatic function, improve mitochondrial and lysosomal quality control, and interrupt inflammatory positive feedback loops. Further clarification of mechanisms at the cell type level, effects that vary according to disease stage, and translational biomarkers will be critical for developing effective interventions against neurodegenerative diseases driven by metabolic and inflammatory dysregulation.
